# Synergy effects and it’s influencing factors of China’s high technological innovation and regional economy

**DOI:** 10.1371/journal.pone.0231335

**Published:** 2020-05-20

**Authors:** Longwu Liang, Zhen Bo Wang, Dong Luo, Ying Wei, Jingwen Sun

**Affiliations:** 1 Institute of Geographic Sciences and Natural Resources Research, Chinese Academy of Sciences, Beijing, China; 2 College of Resources and Environment, University of Chinese Academy of Sciences, Beijing, China; 3 College of Economics and Management, Nanjing University of Aeronautics and Astronautics, Nanjing, China; 4 Torch High-Tech Industry Development Center Ministry of Science & Technology, Beijing, China; 5 College of Geography and Environmental Science, Zhejiang Normal University, Jinhua, Zhejiang, China; Institute for Advanced Sustainability Studies, GERMANY

## Abstract

The Chinese government adheres to the innovation driven strategy and emphasizes that technological innovation is the strategic support for improving social productivity and comprehensive national strength. This paper discusses the mechanism of technological innovation and regional economic co-evolution, and constructs an index system to assess them based on the principles of synergy and systematics. The authors use a dynamic coupling model to study the law of the cooperative evolution of composite systems and geo-detector methods to reveal the main factors controlling the degree of coordination among them. The results show that the total factor productivity of China’s high-tech industry showed a "W"-type trend of change from 2006 to 2016, and the other indices exhibited a volatile trend. The total factor productivity, technical efficiency, scale efficiency, pure technical efficiency, and technological progress increased by 37%, 13.3%, 3.9%, 9%, and 20.8%, respectively. There was a significant spatial difference in changes in total factor productivity, forming a core-edge spatial pattern with the middle and upper reaches of the Yangtze River as the center of concentration. Most of China’s technological innovation and regional economic complex systems were in a state of interactive development from 2007 to 2016, except in the three northeastern provinces of Zhejiang, Shanghai, and the western part of the country. The degree of coupling of the other provinces showed an increasing trend, and the overall degree of coupling exhibited the spatial pattern of Central > Eastern > Western > Northeastern. The three most influential factors for the degree of coupling of China’s provincial complex system were the gross domestic product, efficiency of technological innovation, and expenditure on research and development, whereas the three most important factors affecting the degree of coupling of complex systems were the efficiency of technological innovation, gross domestic product, and number of high-tech enterprises as well as research and development personnel, respectively, in the eastern, central, western, and northeastern regions. Finally, the paper puts forward the suggestions of regional innovation driven coordinated development, technology innovation and regional economic coordinated development, in order to provide reference for the high-quality economic development of developing countries.

## 1. Introduction

The 19th National Congress of China proposed an innovation-driven development strategy, emphasizing that technological innovation provides strategic support for improving social productivity and overall national strength, and thus must be placed at the core of the overall development of the country [1]. Schumpeter [2] claimed that technological innovation is at the core of modern economic growth. Romer [3] proposed that technological progress generated by technological innovation is an endogenous variable of economic growth. Since the beginning of the 21st century, a new round of scientific and technological revolution has brought about clusters of innovation and breakthroughs in the fields of energy, materials, information, agriculture, oceanic research and space, and their major foundations and intersections [4]. Technological innovation has become the main theme of global economic and social development. China is in a period of major transition from an investment-driven, scaled-up and export-oriented mindset to an innovation-driven, quality-growth-oriented and market-led system [5]. It needs to fully implement industrial transformation and technological innovation, and promote the regional economy to a more harmonious and sustainable path of development through high-tech industries. Therefore, it is urgent to take China’s province as a sample to measure the efficiency of technological innovation of high-tech industry, and to comprehensively evaluate the synergistic development effect of the high-tech industry technological innovation and regional economic composite system and its influencing factors.

The high-tech industry, as a knowledge-intensive industry [6], is an important symbol of the comprehensive strength of the regional economy [7], and can indirectly promote its development through a spillover effect on traditional industries [8]. In recent years, scholars have focused on the green supplier selection in high-tech industries [9], industrial mergers and acquisitions [10–11], and for the assessment of the innovation offered by high-tech products [12–13]. The level of development of high-tech industries in developed countries is higher than in China, and their industrial income is mainly affected by research and development investment [14], technology procurement, market structure, and scale of enterprise [15]. The efficiency of the innovation of China’s high-tech industry is on the rise, but its overall level of development remains low [16]. The efficiency of urban green economy in China shows a step-by-step decreasing law in the East [17], the Central and the East, and the coordination effect between it and urbanization shows a significant upward trend [18]. At present, regional differences in the technological innovation in China’s high-tech industries are prominent, and the level of innovation in the east is significantly higher than that in the central and western regions [19]. The organizational learning [20], the growth of government research and development expenditures [21] have a significant positive effect on the efficiency of technological innovation in the high-tech industries. Specifically, the China’s technological innovation and energy efficiency of these industries exhibit a low level of coordinated development [22]. The coupling relationship between technological innovation in the context of Chinese universities and innovation in the high-tech industry shows a spatial distribution pattern of "high in the east and low in the west" [23]. The high-tech service industry has a long-term equilibrium relationship with the high-tech industry, and the former is conducive to the development of the latter [24]. However, there are few literatures to explore the synergistic development effect of high-tech industry and regional economy and to analyze its influencing factors scientifically.

Technological innovation is the core driving force of high-tech industries, and can help cope with environmental and socio-economic problems [25] to achieve sustainable economic and social development [26] as well as green growth [27]. The efficiency of technological innovation refers to the efficiency of transformation of the input and output of technological innovation—for example, increasing output while maintaining the level of input of innovation, or reducing investment in technological innovation under the condition of ensuring the level of output of innovation [28]. It is used to characterize the capability for technological innovation of a region or an enterprise. In this research, the methods of evaluation used included network analysis (ANP) [29] and data envelopment analysis [30]. They examine the laws of changes to the time and sequence of the efficiency of technological innovation from the perspective of data statistics. The Malmquist index is applied to study trends of dynamic efficiency. This paper combines it with the ArcGIS spatial analysis model to more accurately reveal the temporal and spatial patterns, and variations in the regional efficiency of technological innovation. The coupling coordination degree model can not only measure the coordinated development level of high-tech industrial technology innovation and regional economy, but also reflect the level of high-tech industrial technology innovation and regional economy. Combined with the geographic detector model, it can further analyze the impact mechanism of the composite system of technology innovation and regional economy.

In constructing an index system to assess regional technological innovation and regional economic co-evolution, this paper considers the dynamic interaction between China’s provincial technological innovation and space for economic development to minimize the efficiency of “unexpected output value.” Based on the Malmquist index, the total factor productivity and its decomposition index for China’s provincial high-tech industry from 2006 to 2016 were examined. Based on the mechanism of co-evolution of the high-tech industry and the regional economy, a coupling model for a complex of the two is constructed and the law of the synergistic evolution between them is discussed. The geo-detector method is then to analyze the factors influencing composite systems in different regions of China.

## 2. Co-evolution of technological innovation in high-tech industries and regional economy

### 2.1. Path of innovation of high-tech industry

**Fig 1:**
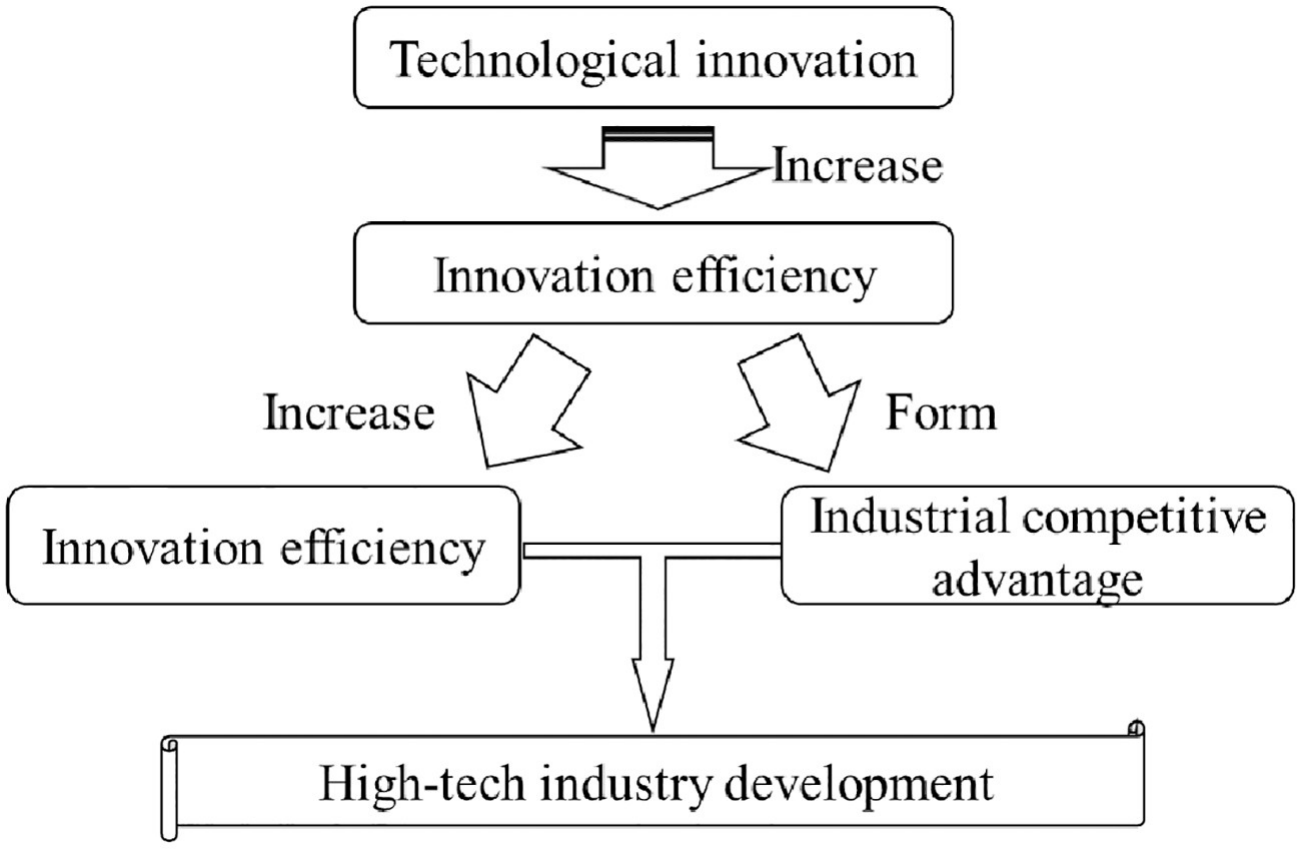
Positive evolution path of technological innovation in high-tech industries.

In the development of the high-tech industry, technological innovation and industrial efficiency have complementary functions. Improving the level of industrial technological innovation can improve production efficiency, increase resource utilization, and ease supply pressures from natural resources and social resources to increase profit. Moreover, the expenditure saved by improving industrial efficiency can be used for the high-tech science and technology infrastructure, such as for the construction of scientific research institutions and universities, investment in high-tech enterprises, and training high-level talent in universities and enterprises. In addition, under fierce market competition, enterprises with differentiated labor productivity abide by the principle of the survival of the fittest, thereby optimizing the spatial allocation of industrial resources and promoting the rapid development of high-tech industries [31]. Overall, the technological innovation of high-tech industries follows the cycle shown in Fig 1.

### 2.2. Action path of technological innovation and regional economic growth

**Fig 2:**
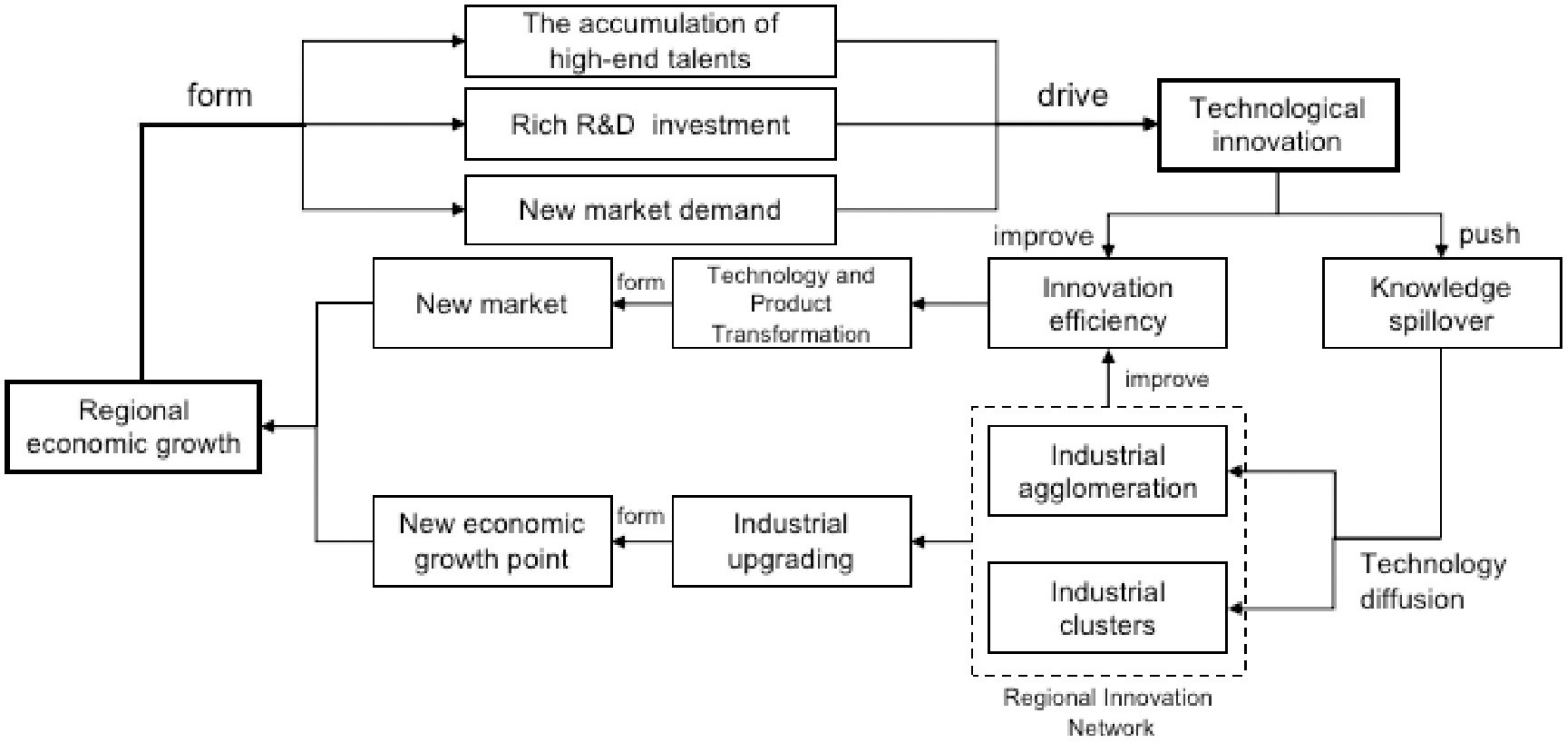
The action path of technological innovation and regional economy.

Technological innovation and economic growth are both causal interactions, promote and restrict each other, and form a two-in-one relationship. In order to establish a more comprehensive compound co-evolution system, the path between them should be clarified. Technological innovation achieves regional economic growth through two paths (Fig 2): First, technological innovation enables highly efficient transformation of technological results by improving the efficiency of enterprise innovation, thereby creating better business value for the technology or product, providing it with new markets, and ultimately achieving economic growth. Second, technological innovation drives the aggregation and cluster development of regional industries through knowledge spillovers and technology diffusion, forming a regional innovation network, thereby promoting industrial upgrading and forming new economic growth points [32]. Talents and R&D investment are considered to be the two most important conditions for technological innovation [33]. Exactly, in turn, the growth of the regional economy has led to the accumulation of high-end talents and enriched R&D investment, creating two most important conditions for regional technological innovation. In addition, economic growth has created new market demand, and the three jointly drive the region development of technological innovation.

### 2.3. Mechanism of co-evolution of technological innovation and regional economy

The cooperative mechanism of evolution between regional technological innovation and the composite industrial system is divided into two modes: intra-subsystem evolution, and the evolution of interaction of the composite system. The subsystem consists of a high-tech industrial technological innovation subsystem and a regional economic subsystem. In the former, research and development capital, talent, technology, and other inputs to innovation output such factors as industrial profits and patents after a series of transformations. Some of the output of innovation is transformed into elements of high-tech industrial enterprises and scientific research institutions to form a higher-quality infrastructure for the high-tech industry that can support innovative input–output activities. The internal co-evolution of the regional economic subsystem is similar to the high-tech industrial technological innovation subsystem, except that its economic input and foundation attend more to the participation of such entities as the society, government, and residents, and its economic output is measured by regional GDP, regional export volume, and other indicators.

**Fig 3:**
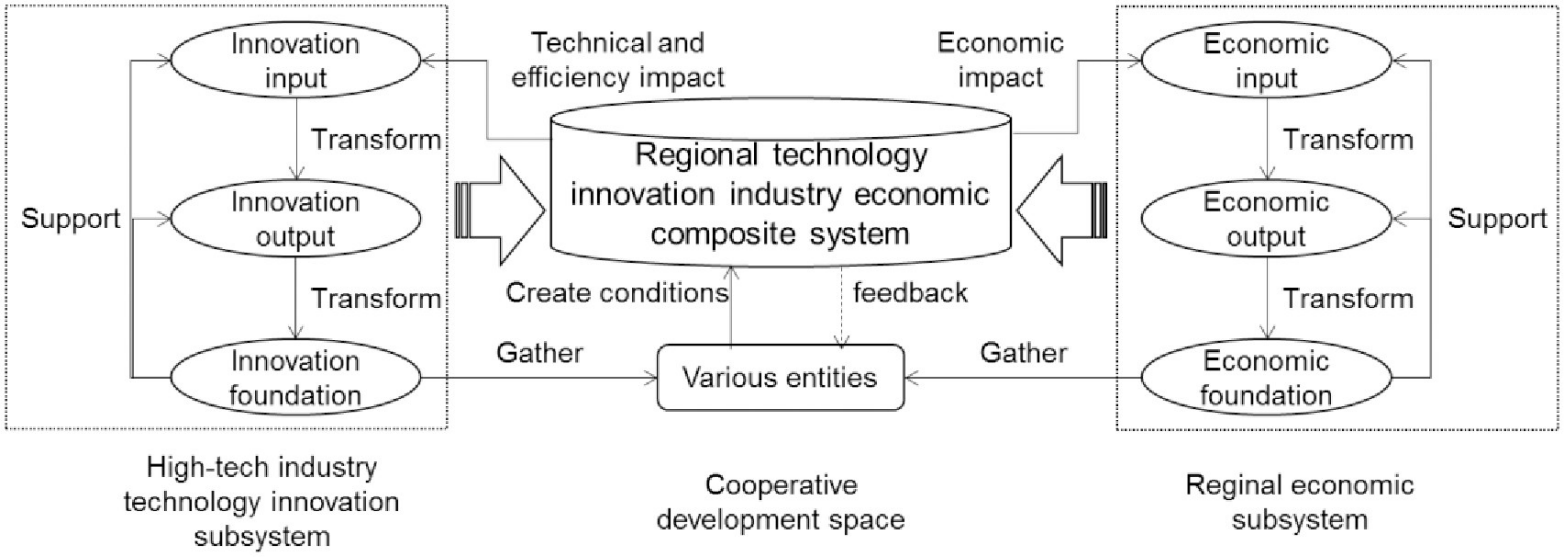
Mechanism of synergistic evolution of technological innovation and regional economic composite system.

The internal transformation and external integration of the two subsystems form an industrial economic composite system of regional technological innovation, and interact to realize the coordinated evolution of the system. The economic foundations of high-tech enterprises, talent, scientific research institutions, and other economic foundations—such as society and household consumption activities—are gathered into various entities that incorporate social and environmental factors, such as science and technology, the market, and policies into a collaborative evolution platform for composite systems. This helps create conditions for interactive activities. In contrast, the collaborative state of the composite system is fed back to various entities so that the foundation of innovation, regional economic foundation, and the coordinated evolutionary environment are continually adjusted to create new conditions for the benign evolution of the composite system [34]. After the combination of input, output, entity, and environment, the goals of the development of the high-tech industry and its economic growth, such as technological progress and efficiency, complete a new round of co-evolution. The overall mechanism of this synergistic evolution is shown in Fig 3.

## 3. Indicator system, data source, and weight

### 3.1. Indicator system and data source

In the research [35–36], most scholars regard high-tech industries and regional economies as independent entities, and establish relevant models to analyze whether they influence each other and why. This paper merges the two systems. Technological innovation is the core power of the high-tech industry economic growth, regional economic development can lay a good environment for technological innovation, support and drive technological innovation, which are inseparable. With reference to the index system used by Si et al. [37], a regional high-tech industrial economic system is constructed that contains a high-tech industrial subsystem and a regional economic subsystem. It can effectively analyze the interaction between the two systems and further explore the impact mechanism of regional high-tech industrial economic composite system. According to the idea of input and output, the subsystem of the high-tech industry is divided into innovation input, innovation output, and innovation development support. The regional economic subsystem is divided into the basic economic level, economic construction input, and economic construction output (Table 1). Among them, considering the data available factors, this paper uses the index “number of college students per 10000” to reflect the level of regional higher education [38].

**Table 1:** Indicator classification and index weights of the high-tech industry subsystem and regional economic subsystem.

Subsystem	Primary indicators and weights	Secondary indicators	Objective weight of secondary indicators	Subjective weight of secondary indicators	Comprehensive weight of Secondary indicator
Technological innovation	Innovation input (0.312)	Number of research and development personnel	0.128	0.153	0.141
		research and development expenditure	0.287	0.247	0.267
		New product development expenditure	0.194	0.291	0.243
		Investment amount	0.168	0.193	0.181
		Number of newly started projects	0.223	0.116	0.170
	Innovation output(0.435)	High-tech industry total profit	0.376	0.328	0.352
		High-tech product exports	0.244	0.198	0.221
		New product sales revenue	0.177	0.257	0.217
		Number of valid invention patents	0.203	0.217	0.210
	Innovation development Support(0.253)	Number of scientific institutions	0.427	0.381	0.404
		Number of college students per 10,000	0.308	0.266	0.287
		Number of high-tech companies	0.265	0.353	0.309
Regional economy	Basic economic level(0.395)	Per capital consumption expenditure of residents	0.383	0.365	0.374
		The total retail sales of social consumer goods	0.274	0.236	0.255
		Resident savings deposit	0.343	0.399	0.371
	Economic construction input(0.224)	Fixed asset investment of the entire society	0.272	0.299	0.286
		Foreign direct investment	0.402	0.342	0.372
		Financial expenditure	0.326	0.359	0.343
	Economic construction output(0.381)	GDP	0.321	0.372	0.347
		Revenue	0.304	0.343	0.324
		Total export	0.375	0.285	0.330

Owing to a lack of data from Tibet, Hong Kong, Macao and Taiwan, the paper selected data for the 30 provinces (municipalities and autonomous regions) of China from 2006–2016 as research sample. Missing data for a few provinces were supplemented by exponential translation. Research data were mainly from the 2007 2017 China High-tech Industry Statistical Yearbook (http://navi.cnki.net/knavi/YearbookDetail?pcode=CYFD&pykm=YZGGJ), China Statistical Yearbook (http://navi.cnki.net/knavi/YearbookDetail?pcode=CYFD&pykm=YINFN), China Financial Yearbook (http://navi.cnki.net/knavi/YearbookDetail?pcode=CYFD&pykm=YXCVB), China Science and Technology Statistical Yearbook (http://navi.cnki.net/knavi/YearbookDetail?pcode=CYFD&pykm=YBVCX).

### 3.2. Data standardization and weight

This paper uses the dimensionless approach to eliminate the magnitude of the data. In the process of data standardization, the linear proportional method [39] can ensure that the difference ratio of objects of the same indicator is constant, which can highlight the difference and make the results of the input–output efficiency more accurate. This method was used to standardize the processing of data for high-tech industrial subsystems. The extreme value processing method [40] can reflect the overall level of all decision-making units in each index. Standardized processing was used to determine the degree of coupling between data for the two subsystems of technological innovation and regional economy. The indicators used were all forward-type indicators, and both subsystems were subjected to forward-type standardization processing.

Subjective and objective comprehensive weightings were carried out by the Analytic Hierarchy Process and entropy method. The results are shown in Table 1. In the process of subjective empowerment, the one–nine scale method was used for the opinions of 40 experts of the Chinese Academy of Sciences at Tsinghua University, Peking University, Beijing Normal University, and other scientific research units and a judgment matrix was constructed, A consistency check of the matrix was performed. The formula for the entropy method is common and is not repeated here.

## 4. Research methods

### 4.1. Malmquist productivity index

In the analysis of technical efficiency, each province of China was regarded as an independent decision-making unit. The DEA-based Malmquist productivity index proposed by Fare was used to estimate changes in the total factor productivity of China’s high-tech industries. The innovation output indexes are the high-tech industry total profit, high-tech product exports, new product sales revenue, number of valid invention patents, and the innovation input indexes are the number of research and development personnel, research and development expenditure, new product development expenditure, investment amount, number of newly started projects (Table 1). This method can decompose the malmquist productivity index into pure technical efficiency index, scale efficiency index and technological progress index, and analyze the evolution process of technological innovation in more detail [41]. From period *t* to period *t* + 1, the Malmquist index measurement of the growth in total factor productivity can be expressed as:

$$M_{0}(x_{t},y_{t},x_{t+1},y_{t+1})={\left[\frac{D_{0}^{t+1}(x_{t+1},y_{t+1})}{D_{0}^{t+1}(x_{t},y_{t})}\times \frac{D_{0}^{t}(x_{t+1},y_{t+1})}{D_{0}^{t}(x_{t},y_{t})}\right]}^{1/2}$$ (1)

In the above formula,$D_{0}^{t}(x_{t},y_{t}),D_{0}^{t}(x_{t+1},y_{t+1})$ are the distance functions of periods *t* and *t* + 1, respectively, under the reference of technology *T*^*t*^ in period *t*, and $D_{0}^{t+1}(x_{t},y_{t}),D_{0}^{t+1}(x_{t+1},y_{t+1})$ are respectively the distance functions of periods *t* and *t* + 1 under the reference of technology *T*^*t*+1^ in period *t* + 1 [42]. The malmquist productivity index can be decomposed into two parts: technical change (techch) and technical efficiency change (effch). The technical efficiency change (effch) can be further decomposed into scale efficiency change (sech) and pure technical efficiency change (pech). Eq (1) can be rewritten as:

$$M_{0}\left(x_{t},y_{t},x_{t+1},y_{t+1}\right)=\underset{effch}{\underset{\text{sech}}{\frac{S_{0}^{t}\left(x_{t},y_{t}\right)}{S_{0}^{t}\left(x_{t+1},y_{t+1}\right)}}\times \underset{pech}{\frac{D_{0}^{t}\left(x_{t+1},y_{t+1}\right)}{D_{0}^{t}\left(x_{t},y_{t}\right)}}}\times \underset{techch}{{\left[\frac{D_{0}^{t+1}\left(x_{t+1},y_{t+1}\right)}{D_{0}^{t+1}\left(x_{t},y_{t}\right)}\times \frac{D_{0}^{t}\left(x_{t+1},y_{t+1}\right)}{D_{0}^{t}\left(x_{t},y_{t}\right)}\right]}^{1/2}}$$ (2)

In the above formula, the first term represents the change in scale efficiency, the second represents the change in pure technical efficiency, the third term represents technical change, and the Malmquist productivity index (tfpch) is represented by these three terms. Sech>1 indicates that the scale of agglomerated investment in the high-tech industry was optimized and scale efficiency improved; pech>1 indicates that the efficiency of resource utilization and configuration structure improved; techch>1 indicates the comprehensive productivity of the high-tech industry improved. Conversely, this means that the efficiency degraded when the above indicator was smaller than one [43].

### 4.2. Dynamic coupling model

The high-tech industrial subsystem and regional economic subsystem appear to operate independently of each other but the correlation between them is strong, and they interact on the whole and in part. This paper uses the dynamic coupling model [44], first constructed a series of equations of the dynamic evolution of composite systems (30 equations in total, omitted here because of limitations of space) based on the idea of the Bertalanffy general system theory [45]. The above equations were separately derived to obtain the evolutionary velocity equation of the composite system. The ratio of the rate of the evolution of the composite system to that of the two subsystems in each region was then obtained by using time as a time series variable. The degree of systemic coupling in each region in each year was then obtained by using the inverse tangent function. The degree of systemic coupling reflected the ratio of the speeds of evolution of the two subsystems.

The dynamic coupling model considers the integrity and dynamics of the system, elemental interactions between the high-tech industrial subsystem and the regional economic subsystem, and dynamic changes in the subsystems over time to reflect the law of evolution of composite systems.

#### 4.2.1 Equation of evolution of composite system based on Bertalanffy general system theory

The coupling process between the high-tech industry and the regional economic subsystems can be expressed through the quantification of the two and their process of interaction. A system coupling process model was constructed by referring to the idea of system evolution in general system theory. The processes of change in the high-tech industrial subsystem and regional economic subsystem are nonlinear, and models of their evolution can be expressed as:

$$\frac{dx(t)}{dt}=f(x_{1},x_{2},\ldots ,x_{m}),j=1,2,\ldots ,m$$ (3)

In the formula, *f* is *x*_*j*_ nonlinear function obtained by Excel fitting (we can judge the reasonableness of the fitting function based on the degree of fit). Because the stability of a nonlinear system depends on the nature of an approximate system feature root [46], under the premise of ensuring the stability of motion, the above nonlinear system was obtained after expanding it in the Taylor series near the origin and omitting the highest order:

$$\frac{dx(t)}{dt}=\sum_{j=1}^{m}w_{j}x_{j},j=1,2,\ldots ,m$$ (4)

*te*,*se* represent the total high-tech industry and regional economy subsystems (the indicators in the Table 1), and their relationships are as follows:

$$\begin{array}{l} f(te)=\sum_{j=1}^{m}w_{1j}x_{1j},j=1,2,\ldots m(m=15)\\ f(se)=\sum_{j=1}^{m}w_{2j}x_{2j},j=1,2,\ldots ,m(m=10) \end{array}$$ (5)

In the above formula, *x*_1*j*_, *x*_2*j*_ represent elements of the high-tech industry and regional economic subsystem, respectively, and *w*_1*j*_, *w*_2*j*_ represent the weight of each element. According to the Bertalanffy general system theory, the two subsystems form a composite system, and the equation of its evolution can be expressed as:

$$\left\{ \begin{array}{c} A=\frac{df(te)}{dt}=\alpha_{1}f(te)+\alpha_{2}f(se)\\ B=\frac{df(se)}{dt}=\beta_{1}f(te)+\beta_{2}f(se) \end{array}\right.$$ (6)

In the above formula, *α*_1_, *α*_2_ represent the response coefficients of the high-tech industrial subsystem and the regional economic subsystem, respectively, to the evolution of the former. They express the contributions of the state representations of the two subsystems to the rate of evolution of the high-tech industrial subsystem. *β*_1_, *β*_2_ represent the response coefficients of the high-tech industrial subsystem and the regional economic subsystem, respectively, to the evolution of the the latter. They express the contributions of the state representations of the two subsystems to the rate of evolution of the regional economic subsystem. The above parameters indicate the direction and intensity of interactions between the two subsystems, and may all be constant at some stage of system development.

#### 4.2.2 Solving for degree of system coupling based on arctangent function

In the system, the two subsystems interact. Any change in one causes changes in the entire system. Under these influences, speed of evolution is expressed as:

$$V_{A}=\frac{dA}{dt},V_{B}=\frac{dB}{dt}$$ (7)

Because the system contains only two elements *f*(*te*) and *f*(*se*), when they are coordinated, the entire system is coordinated. The speed of evolution *V* of the system can be seen as a function of *V*_*A*_ and *V*_*B*_, and thus *V* = *f*(*V*_*A*_, *V*_*B*_). Then, we can study the coordinative relationship of the system *f*(*te*) and *f*(*se*) through changes of *V* when using *V*_*A*_ and *V*_*B*_ as control variables. Because the regional economic subsystem evolved more quickly than the high-tech industrial subsystem, its trajectory was an ellipse with the following relationship:

$$\text{tan}\;\theta =\frac{V_{A}}{V_{B}},\theta =\text{arctan}\frac{V_{A}}{V_{B}}$$ (8)

In the formula, *θ* represents the degree of coupling between the subsystems. We can judge the state of evolution of the system and the degree of coupling between the subsystems according to *θ*. The degree of coupling represents the variation between coordinate quadrants, which is a continuous cycle process. *n* takes an integer.

In an evolutionary cycle, the entire system underwent four stages: interactive development, system degradation, system collapse, and system regeneration [47].

1. 0° + 2*πn* ≤ *θ* < 90° + 2*πn* indicates that the high-tech industry and the regional economic system developed interactively. *θ* = 0° + 2*πn* is the starting point of the high-tech industry and regional economic system coordination. 0° + 2*πn* < *θ* < 45° + 2*πn* indicates the coordinated development of the two systems, *θ* = 45° + 2*πn* indicates that the speed of development of the two subsystems is equal, and is the best position for coordinated development. 45° + 2*πn* < *θ* < 90° + 2*πn* indicates that the development of high-tech industries is constrained by regional economic development.
2. 90° + 2*πn* ≤ *θ* < 180° + 2*πn* indicates that the high-tech industry and the regional economic system were in a state of degradation. The regional economic development began to experience negative growth while the development of high-tech industries slowed, and the entropy of the entire system increased.
3. 180° + 2*πn* ≤ *θ* < 270° + 2*πn* indicates that the high-tech industry and regional economic system were in a state of disintegration, and experienced negative growth. *θ* = 180° + 2*πn* indicates that the regional economic growth rate was zero. With continuous increase in θ, the negative growth rate of high-tech industries continued to decrease, eventually reaching zero, and the negative regional economic rate of growth increased.
4. 270° + 2*πn* ≤ *θ* < 360° + 2*πn* indicates that the high-tech industry and regional economic system were in the process of regeneration. The high-tech industrial system was beginning to evolve, the economic recession of the regional economic system was subsiding, and they entered a new evolutionary cycle.

### 4.3. Geographic detector method

The geo-detector method was developed by Chinese scholar Wang Jinfeng and was first used to detect the mechanism of influence of geospatial factors on risk of disease [48]. This method does not require too many assumptions and can overcome the limitations of statistical variable processing. The method can be used to identify influential factors, and can help analyze their interaction with the dependent variables. It is an effective tool for studying the driving mechanisms of complex geographical factors [49], and is widely used to study human and natural factors.

#### 4.3.1 Factor detector

The core idea of the factor detector is to determine whether changes in an environmental factor and a geographical occurrence have significant spatial consistency. If they are consistent, this indicates that the environmental factor is decisive for the occurrence and development of the geographical occurrence.

$$q_{DU}=1-\frac{1}{n\sigma_{U}^{2}}\sum_{i=1}^{m}n_{D,i}\sigma_{U_{D,i}}^{2}$$ (9)

*q*_*DU*_ is the impact detection index of the influential factors on the degree of coordination of urbanization and the ecological environment composite system, *n* is the number of research units, *n*_*D*,*i*_ is the number of samples of the second-level research unit, *m* is the number of secondary research units, $\sigma_{U}^{2}$ is the variance of the degree of coordination of the urban agglomeration complex system, and $\sigma_{U_{D,i}}^{2}$ is the variance in the degree of coordination between the subsystems. Assuming $\sigma_{U_{D,i}}^{2}\neq 0$, the model is established. The range of values of *q*_*DU*_ is [0,1]. *q*_*DU*_ = 0 indicates that the spatial distribution of the degree of coordination of the composite system was not driven by the influential factors. The larger the value of *q*_*DU*_ was, the greater the influence of the natural factors was on the degree of coordination of the composite system. The different categories of the influence degree (Table 2) were divided referring to the relevant literature [50] and according to the range of influence coefficient.

**Table 2:** Classification of impact levels.

Impact coefficient	0.8 ≤ *q* ≤ 1	0.7 ≤ *q* < 0.8	0.6 ≤ *q* < 0.7
Impact category	Extreme impact	Greatly impact	Large impact
Impact coefficient	0.5 ≤ *q* < 0.6	0.3 ≤ *q* < 0.5	0 ≤ *q* < 0.3
Impact category	General impact	Smaller impact	Weak impact

#### 4.3.2 Interaction detector

**Fig 4:**
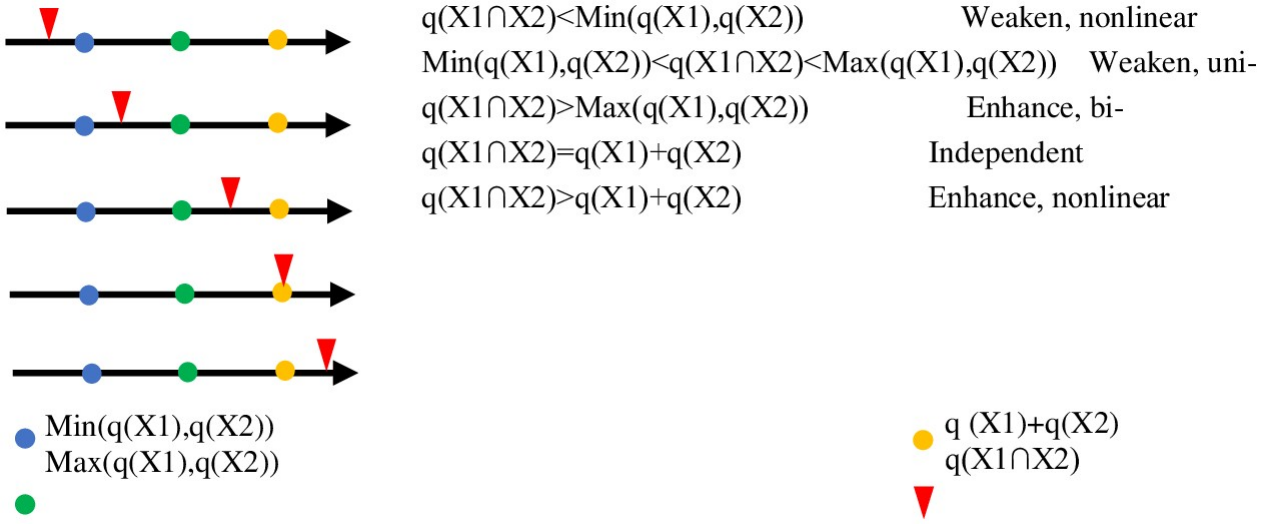
Redefined interaction relationships.

The detection of interaction can be used to quantitatively characterize the relationship between the influential factors in terms of the degree of coordination of the composite system. For example, for factors A and B affecting the degree of coordination of the composite system, a new layer C is formed by spatially superimposing A and B. The attributes of C are determined by A and B together. By comparing the influence of layers A and B with that of layer C, the strength of the influence of the interaction of the two factors on the spatial pattern of the degree of coordination of the composite system can be determined (Fig 4).

## 5. Results

### 5.1. Characteristics of dynamic temporal and spatial evolution of efficiency of technological innovation in provincial high-tech industry

In this paper, the linear proportional method was used to measure the indices of the high-tech industrial subsystem of China’s 30 provincial-level administrative regions from 2006 to 2016, and the DEA model was used to measure the provincial high-tech industry’s Malmquist and decomposition indices from 2006 to 2016 (with 2006 as the base year, the five index values were all one). This was intended to reveal the characteristics of the dynamic evolution of the efficiency of China’s provincial technological innovation in time and space.

#### 5.1.1 Temporal characteristics

The total factor productivity reflects the level of efficiency of the technological innovation of high-tech industries, that is, the input–output ratio of innovation in high-tech enterprises. From 2006 to 2016, China’s high-tech industry developed rapidly, the scale of industry expanded, the level of technology improved, and the total factor productivity increased by 37%. Technical efficiency increased by 13.3%, scale efficiency by 3.9%, pure technical efficiency by 9%, and technological progress increased by 20.8%. In recent years, the state has invested significant funds to promote technological innovation. The growth in technological efficiency is the core force driving the growth of the total factor productivity in China’s high-tech industries, and both have exhibited a trend of a “spiral rise.” The improvement in technical efficiency is mainly owing to the increase in pure technical efficiency, which in turn is the result of a rapid improvement in the capability for technological innovation of scientific research institutions and high-tech enterprises. From 2006 to 2011, technical efficiency declined, and technological progress grew rapidly. China’s high-tech industry is an export-oriented economy; due to the financial crisis, technical efficiency decreased, and the crisis awareness of companies has promoted rapid technological improvement. The growth of technological progress has since been slow whereas technical efficiency is growing rapidly. With an emphasis on the high-tech industry in the 12th Five Year Plan and the proposal of the national One Belt, One Road strategy, high-tech industrial technology has been continually optimized and its efficiency improved to yield a snowball effect.

**Fig 5:**
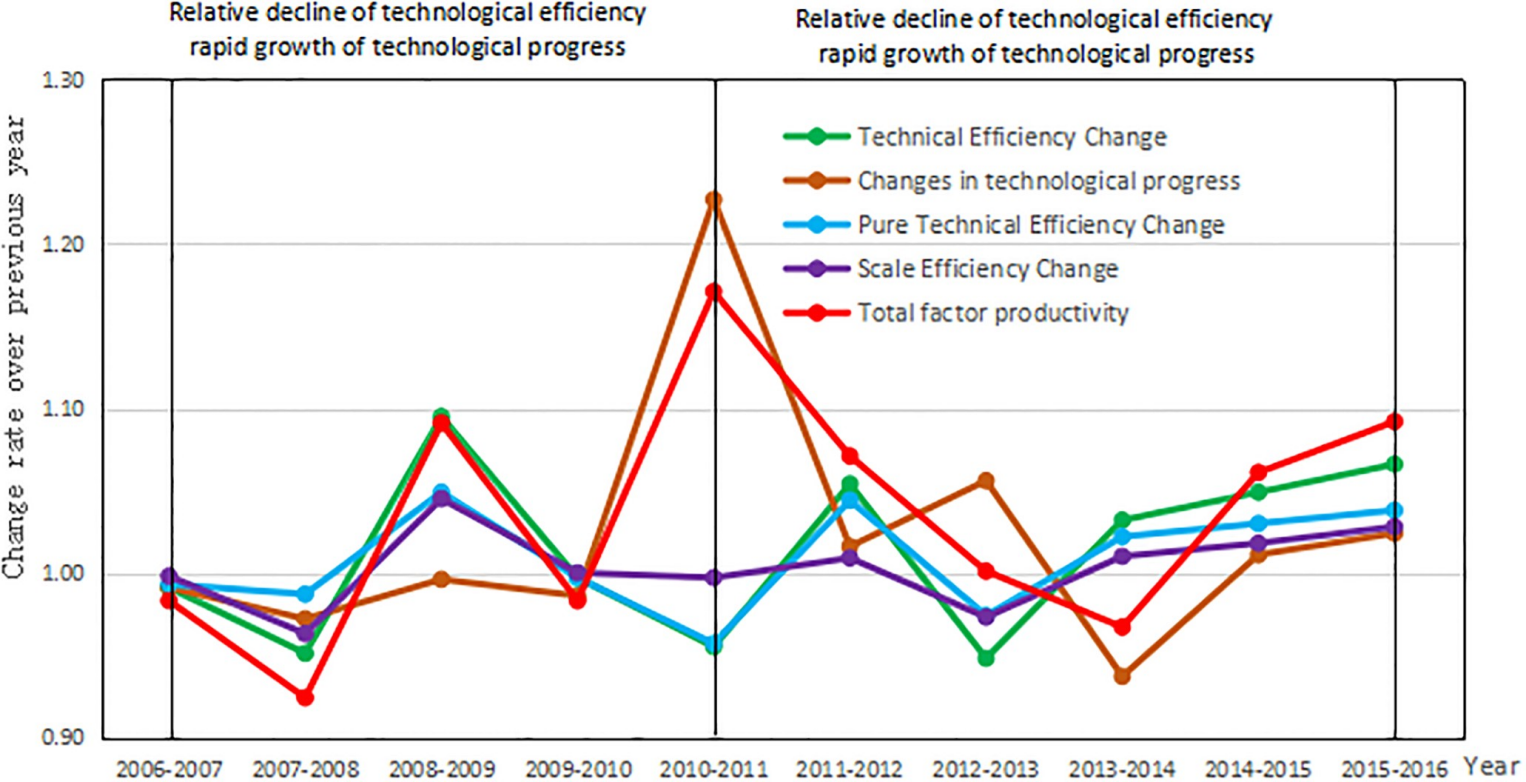
Time series evolution of the efficiency of technological innovation of China’s high-tech industry from 2006 to 2016.

In terms of evolutionary trends, the total factor productivity of China’s high-tech industry showed a composite W-type trend of change, and the other indices followed a wave-like law of evolution from 2006 to 2016. The technological progress index exhibited the largest fluctuation and pure technical efficiency yielded the smallest. Fluctuations in technical efficiency and scale efficiency were moderate, and were consistent (Fig 5). In terms of total factor productivity, except in 2010 and 2014, the DEA state of efficiency (DEA efficiency shows that the index is greater than or equal to one) was achieved in all other years, and peaked in 2011. This is because the national 12th Five Year Plan was officially released in 2011, and called for the development of high-tech industries, which has since enabled the industry to develop at an unprecedented rate. The trends of development of technical and scale efficiency have been highly consistent, and there was high coordination between the efficiency of resource allocation as represented by technical efficiency and the design of the management system as represented by scale efficiency. This indicates that the national incentive and support policies enhance the technical efficiency of the high-technology industry.

#### 5.1.2 Spatial characteristics

**Fig 6:**
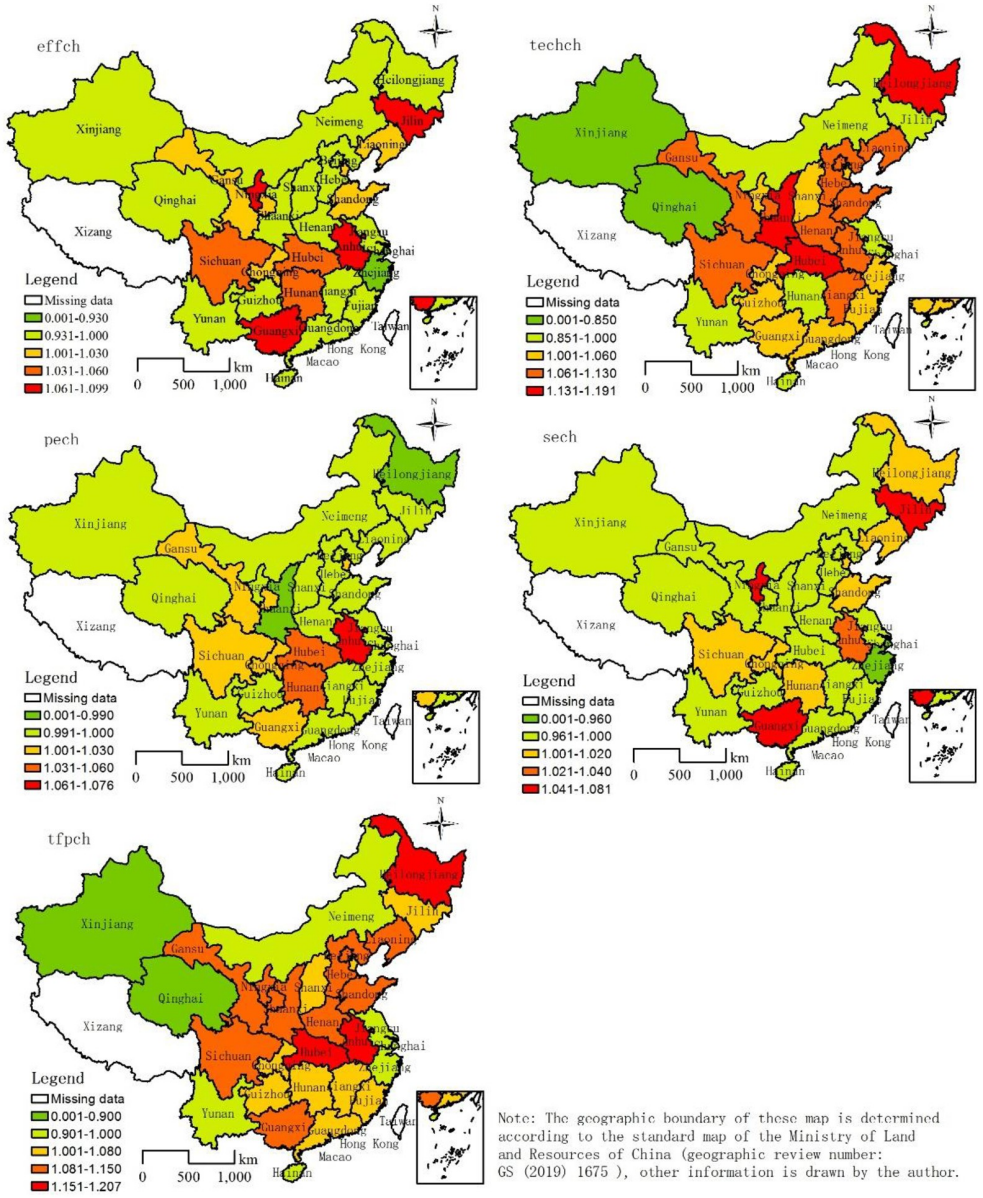
Spatial distribution of annual average of total factor productivity and its decomposition index for China’s high-tech industry from 2006 to 2016.

There was a significant spatial difference in changes in total factor productivity in high-tech industries, forming a core-edge pattern with the middle and upper reaches of the Yangtze River as the center of concentration. There were eight declines in total factor productivity in the 30 provinces (municipalities, autonomous regions) and 22 rises (Table 3) in 2006–2016. The total factor productivity of economically backward areas, such as the northwestern and northern areas, and regions of Yunnan, economically developed areas such as Jiangsu, Zhejiang, Shanghai, and the southern coastal areas declined significantly. The total factor productivity of the middle and upper reaches of the Yangtze River, the Yellow River Basin, and the three northeastern provinces showed a clear upward trend, with the middle and upper reaches of the Yangtze River rising the most significantly (Fig 6). The decline in total factor productivity in Jiangsu, Zhejiang, Shanghai, and the southern coastal areas stemmed from their lower technical efficiency than the national average (1.008), less than one. The economies were more developed, the technological levels were higher, more technical talent was available, and the conditions for innovation and entrepreneurship were better, but factor input far exceeded other regions, and resource allocation and utilization efficiency was low in the above areas. The decline was due to slower technological progress than the national average (1.043) in the northwest and northern area, and regions of Yunnan. The backwardness of regional transportation and poor economic conditions hindered the development of high-tech industries, with fewer technically qualified people, and scientific and technological activities. It is worth noting that the total factor productivity of high-tech industries in the three northeastern provinces was higher than the national average (1.052). In recent years, the state has launched a revitalization strategy for the old industrial bases in the northeast. The government at all levels has promoted institutional reforms to drive industrial transformation with a focus on large and medium-sized enterprises. The cumulative effect of factor inputs promotes technological advancement in high-tech industries. However, only a comparative advantage in the northeastern region was observed compared with developed regions in eastern China, and there was still a big gap in terms of absolute superiority.

**Table 3:** Classification of total factor productivity and its decomposition index for China’s high-tech industry in 2006–2016.

Trend	Technical efficiency change (Effch)	Technological change (Techch)	Pure technical efficiency change (Pech)	Scale efficiency change (Sech)	Total factor production rate (Tfpch)
Provinces and cities experiencing a rise	Shandong, Liaoning, Tianjin, Chongqing, Gansu, Hubei, Sichuan, Hunan, Ningxia, Jilin, Guangxi, Anhui	Guangxi, Zhejiang, Guizhou, Tianjin, Fujian, Guangdong, Chongqing, Ningxia, Shanxi, Anhui, Gansu, Jiangxi, Beijing, Shandong, Hebei, Sichuan, Liaoning, Henan, Shaanxi, Hubei, Heilongjiang	Guangxi, Tianjin, Chongqing, Gansu, Sichuan, Hunan, Hubei, Anhui	Tianjin, Heilongjiang, Chongqing, Shandong, Liaoning, Sichuan, Hunan, Anhui, Ningxia, Guangxi, Jilin	Guizhou, Fujian, Guangdong, Hunan, Tianjin, Chongqing, Shanxi, Jiangxi, Jilin, Guangxi, Beijing, Hebei, Gansu, Ningxia, Shandong Shaanxi, Liaoning, Henan, Sichuan, Anhui, Heilongjiang, Hubei
Provinces and cities that remained unchanged	Xinjiang, Qinghai, Inner Mongolia, Guizhou, Fujian, Guangdong, Shanxi, Beijing, Henan	Shanghai, Hainan, Jiangsu	Xinjiang, Qinghai, Inner Mongolia, Yunnan, Jilin, Shanghai, Hainan, Jiangsu, Zhejiang, Guizhou, Fujian, Guangdong, Ningxia, Shanxi, Jiangxi, Beijing, Shandong, Hebei, Liaoning, Henan	Xinjiang, Qinghai, Inner Mongolia, Guizhou, Fujian, Guangdong, Shanxi, Beijing, Henan, Gansu	——
Declining provinces and cities	Zhejiang, Shanghai, Shaanxi, Hainan, Jiangsu Heilongjiang, Yunnan, Jiangxi, Hebei	Xinjiang, Qinghai, Inner Mongolia, Yunnan, Hunan, Jilin	Shaanxi, Heilongjiang	Zhejiang, Shanghai, Hainan, Hubei, Jiangsu, Yunnan, Jiangxi, Hebei, Shaanxi	Xinjiang, Qinghai, Shanghai, Zhejiang, Inner Mongolia, Hainan, Yunnan, Jiangsu

Changes in the technological efficiency of high-tech industries were generally small, and the regions recording an increase exhibited spatial clusters and distributions, forming a group-band distribution pattern with the middle and upper reaches of the Yangtze River as the center. There were nine declines and 21 rises (Table 3) in the technological efficiency of the 30 provinces (municipalities, autonomous regions). The technological efficiency of the economically backward areas, such as the western, central, southern coastal, and northeastern regions, and economically developed areas, such as Jiangsu, Zhejiang, and Shanghai, declined significantly. The technological efficiency of the middle and upper reaches of the Yangtze River, the upper reaches of the Yellow River, and the Bohai Rim region increased significantly, with the most significant increase in the middle and upper reaches of the Yangtze River (Fig 6). The decline in technical efficiency in the western, central, southern, and northeastern regions was due to the decline in pure technical efficiency, lack of investment in regional technological innovation, lack of technical talent, and a low technical level. Taking Heilongjiang as an example of one of China’s old industrial bases, it lags behind other provinces of China in high-tech industrial technological innovation and lacks technical talent. The decline in technical efficiency was due to a decline in efficiency of scale in Jiangsu, Zhejiang, and Shanghai. That is, investment in high-tech industries was poor, and the allocation and management of resources needs to be improved. The increase in technical efficiency occurred mainly due to an increase in scale efficiency in the middle and upper reaches of the Yangtze River, the upper reaches of the Yellow River, and the Bohai Rim region, especially in the western, central, and northeastern provinces. The government at all levels attached importance to the development of high-tech industries and advocated independent innovation of enterprises, showing a trend of innovation. For these reasons, scale efficiency increased significantly.

### 5.2. Analysis of effect of synergistic development of high-tech industry and regional economic composite system

**Fig 7:**
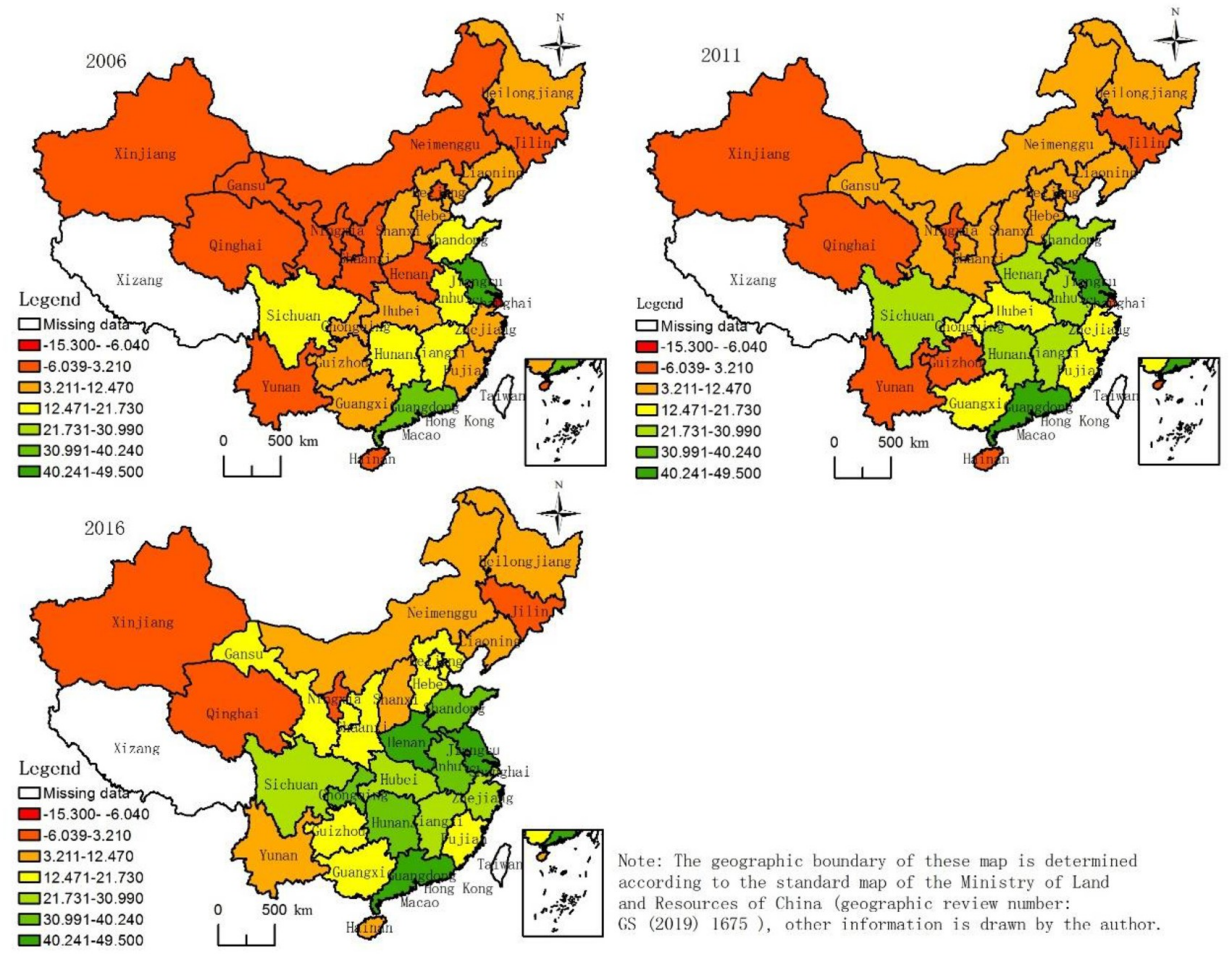
Map of spatial distribution of the degree of coupling between China’s provincial high-tech industry and regional economy.

The linear proportional method and extremum processing method were used to standardize indicators of the high-tech industrial subsystem and regional economic subsystem, and the analytic hierarchy process and entropy method were used to gain the comprehensive weight of the indicators (Table 1). We calculated and summarized the indicators and obtained comprehensive values for the high-tech industrial subsystem and regional economic subsystem in the 30 provinces (cities) from 2006 to 2016. Fitting the years, and values for the provinces and cities in the two systems (representing fitness values (Table 4) of the high-tech industry subsystem and regional economic subsystem), we calculated the degree of reliability (Fig 7) by Eqs (3)–(8).

**Table 4:** Fitness values of coupling model of provincial high-tech industry and regional economic subsystems from 2006 to 2016.

Region			Region			Region		
Beijing	0.995	0.981	Zhejiang	0.986	0.994	Hainan	0.928	0.978
Tianjin	0.974	0.989	Anhui	0.996	0.996	Chongqing	0.999	0.984
Hebei	0.981	0.990	Fujian	0.992	0.991	Sichuan	0.952	0.998
Shanxi	0.921	0.989	Jiangxi	0.978	0.993	Guizhou	0.978	0.989
Neimenggu	0.991	0.987	Shandong	0.984	0.996	Yunan	0.946	0.995
Liaoning	0.922	0.969	Henan	0.995	0.999	Shannxi	0.959	0.991
Jilin	0.942	0.965	Hubei	0.987	0.994	Gansu	0.979	0.974
Heilongjiang	0.939	0.962	Hunan	0.989	0.996	Qinghai	0.968	0.983
Shanghai	0.862	0.988	Guangdong	0.992	0.990	Ningxia	0.940	0.993
Jiangsu	0.983	0.992	Guangxi	0.998	0.970	Xijiang	0.967	0.978

#### 5.2.1 Composite system is in state of interactive development except for a few regeneration stages

The degree of coupling of China’s provincial high-tech industry and regional economic composite system fluctuated in the interval [-15.3, 49.5] from 2006 to 2016, and most values were in the interval [0, 49.5]. That is, the composite system was in a state of interactive development. The high-tech industrial subsystem and regional economic subsystem were both independent and strongly relevant. The two subsystem interacted and evolved at multiple levels and aspects, and a virtuous circle within the composite system was realized based on the synergistic effects of each link, thereby promoting synergy and orderly development among these subsystems.

#### 5.2.2 Degree of coupling of composite systems in provinces showed increasing trend

The degree of coupling of the composite systems decreased year by year in Heilongjiang and Liaoning provinces, while it showed a wave-like trend of change in Guizhou, Ningxia, Jilin, Zhejiang, and Shanghai, and an increasing trend in the rest of the provinces. The development of high-tech industries remains in a crucial period in the western and northeastern provinces. To achieve the goal of generating income with technology requires that the government invest more resources, and that local enterprises attach importance to technological innovation. In 2016, the composite systems of all provinces were in an interactive state of development, which showed that China’s high-tech industry had developed rapidly, and had a strong synergistic and mutually beneficial synergetic effect on the regional economy.

#### 5.2.3 Degree of coupling of composite system showed spatial pattern of Center > East > West > Northeast

The average degrees of coupling of composite systems of the China’s four major plates showed a spatial pattern of Center > East > West > Northeast (Fig 7). The eastern coastal areas underwent strong economic development, and had superior scientific and technological talents to other regions. However, the regional economy and the high-tech industries were strongly independent and did not form a good mutual promotion effect. The economic strength and high-tech industry of the central region were disadvantaged compared with those in the eastern region. However, with the support of the country’s central rise policy, the central region quickly developed in science and technology. This led to a strong synergistic effect between the regional economic system and the high-tech industrial system. Under the influence of the western development policy, the western region made significant progress in its regional economy and invested more resources to support the development of local high-tech industries. However, technological diffusion was difficult in the western region, and the cycle of the conversion effect of the high-tech industrial economy was longer than in the other regions. This led to the weak mutual promotion of high-tech industries and regional economies, which restricted their simultaneous development. The synergy between the high-tech industry and the regional economy weakened year by year in the northeast, where the development of the former could not keep up with that in the latter. An old industrial area of China, its traditional industrial technology could not adapt to modern industry, and local enterprises need to quickly import technological knowledge from abroad to promote technological progress and improve technological innovation.

### 5.3. Factor analysis of degree of coupling of high-tech industry and regional economic composite system

**Fig 8:**
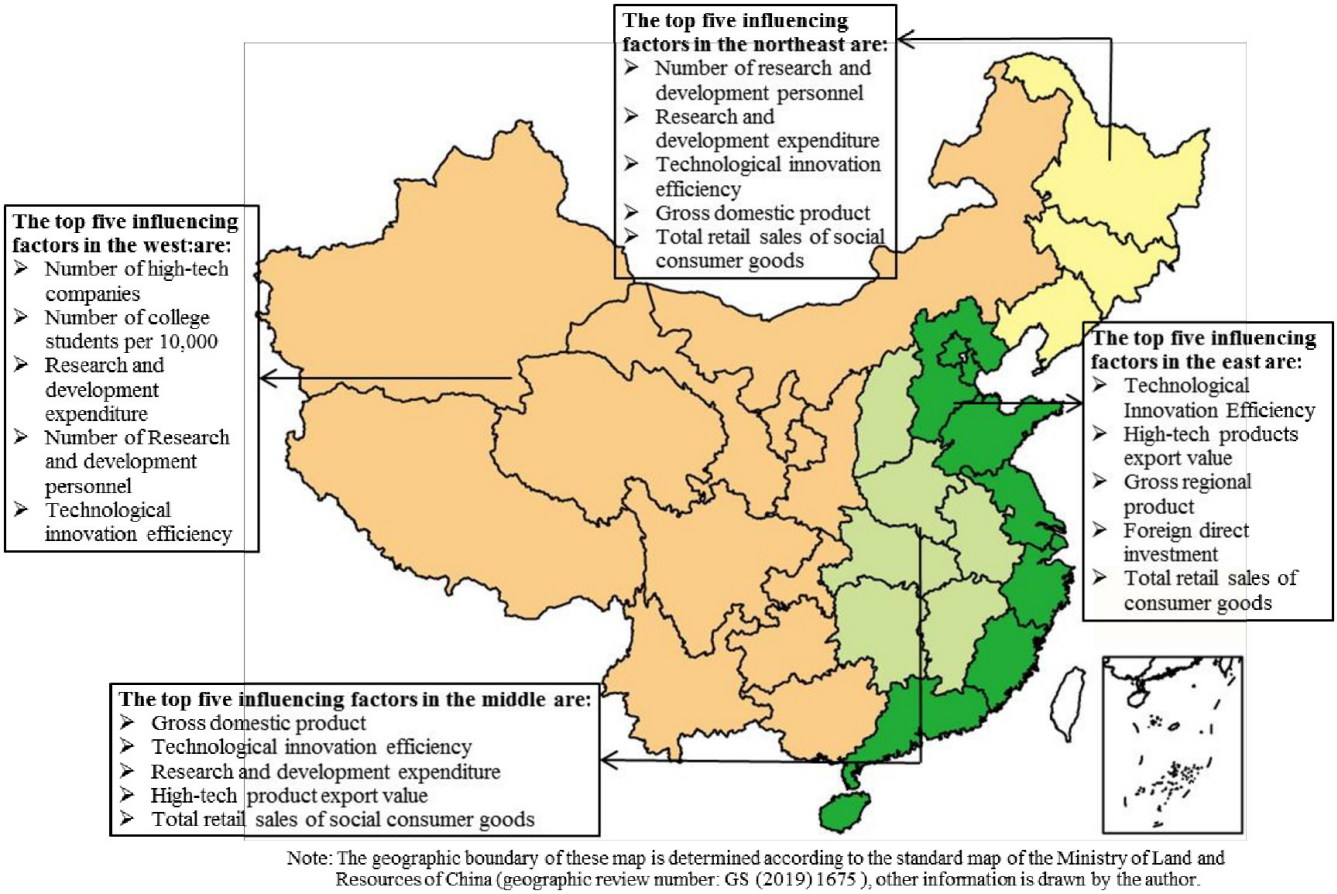
Top five controlling factors of the degree of coupling of composite systems in different regions.

To reveal factors influencing the coupling between technological innovation and the regional economic composite system, the authors selected the efficiency of technological innovation, number of research and development personnel, expenditure on research and development, export value of high-tech products, effective invention patents, number of college students per 10,000, number of high-tech companies, total retail sales of social consumer goods, foreign direct investment, gross domestic product, and total export volume as explanatory variables based on the mechanism of high-tech industry technological innovation and regional economic coordination. The efficiency of technological innovation is the total factor productivity of high-tech industries. The authors used the geographic detector method to explore the main factors influencing the degree of coupling of complex systems in different regions (Fig 8, Table 5) and the interaction between influential factors (Table 6).

**Table 5:** Dominant factors in the degree of coupling between the provincial high-tech industry and the regional economy of composite systems.

Region	National			Eastern China			Central China			Western China			Northeastern China		
Type	q value	p value	q order	q value	p value	q order	q value	p value	q order	q value	p value	q order	q value	p value	q order
Technological innovation efficiency	0.819	0	2	0.873	0	1	0.799	0.03	2	0.770	0	5	0.729	0	3
Number of research and development personnel	0.617	0	6	0.469	0.38	9	0.663	0.77	6	0.789	0.04	4	0.825	0	1
Research and development expenditure	0.745	0	3	0.511	0.71	8	0.792	0	3	0.804	0.01	3	0.744	0	2
High-tech product export value	0.478	0	7	0.824	0	2	0.784	0.04	4	0.622	0.02	8	0.558	0.97	8
Effective invention patents	0.431	0.18	10	0.563	0	7	0.503	0.1	8	0.545	0	9	0.618	0	7
Number of college students per 10,000	0.464	0	8	0.411	0	11	0.461	0	9	0.876	0	2	0.515	0.69	9
Number of high-tech companies	0.654	0	5	0.432	0.25	10	0.426	0.76	10	0.879	0	1	0.655	0	6
Total retail sales of social consumer goods	0.693	0	4	0.685	0.01	5	0.685	0	5	0.684	0	7	0.678	0	5
Foreign direct investment	0.451	0	9	0.717	0.02	4	0.548	1	7	0.509	0.02	10	0.362	0.67	10
Gross domestic product	0.859	0	1	0.799	0	3	0.829	0.07	1	0.751	0.01	6	0.716	0.04	4
Total export volume	0.386	0.04	11	0.578	0.22	6	0.332	1	11	0.458	0.71	11	0.341	1	11

**Table 6:** Factors influencing the degree of coupling between provincial high-tech industry and regional economic composite system.

	Technological innovation efficiency	Number of research and development personnel	Research and development expenditure	High-tech product export value	Effective invention patents	Number of college students per 10,000	Number of high-tech companies	Total retail sales of social consumer goods	Foreign direct investment	Gross domestic product	Total export volume
Technological innovation efficiency	0.819										
Number of research and development personnel	0.912EB	0.617									
Research and development expenditure	0.932EB	0.906EB	0.745								
High-tech product export value	0.881EB	0.794EB	0.874EB	0.478							
Effective invention patents	0.922EB	0.913EB	0.838EB	0.905EN	0.431						
Number of college students per 10,000	0.907EB	0.876EB	0.823EB	0.854EB	0.741EB	0.464					
Number of high-tech companies	0.869EB	0.791EB	0.808EB	0.778EB	0.822EB	0.823EB	0.654				
Total retail sales of social consumer goods	0.912EB	0.904EB	0.862EB	0.901EB	0.857EB	0.861EB	0.854EB	0.693			
Foreign direct investment	0.891EB	0.890EB	0.816EB	0.884EB	0.767EB	0.774EB	0.821EB	0.836EB	0.451		
Gross domestic product	0.862EB	0.848EB	0.788EB	0.858EB	0.842EB	0.856EB	0.836EB	0.881EB	0.841EB	0.859	
Total export volume	0.831EB	0.808EB	0.848EB	0.872EN	0.907EN	0.863EN	0.753EB	0.875EB	0.878EN	0.879EB	0.386

#### 5.3.1 Analysis of factors controlling degree of coupling of China’s provincial complex system

The five most influential factors for the degree of coupling between China’s provincial complex system were the gross domestic product, efficiency of technological innovation, research and development expenditure, total retail sales of social consumer goods, and the number of high-tech companies. The coefficient of influence was higher than 0.6, and all passed the significant level test of 0.01. The first two significantly affected the degree of coupling of the composite system. Since the reform and opening up, China’s economy has continued to grow at a pace, and the per capita GDP has increased from $156.4 in 1978 to $8826.99 in 2017, 55.44 times higher [51]. The innovation capability of its provinces has also improved significantly. However, there is a wide spatial difference between the eastern and western regions, with a significant Matthew’s effect. Expenditure on research and development has a significant impact on the degree of coupling of composite systems. In recent years, China’s expenditure on this has increased constantly. It ranked second in the world on this measure for the first time in 2013. The gap between the total input in China and the US is decreasing year by year. The intensity of input reached the level of moderately developed countries in 2017. The total retail sales of social consumer goods and number of high-tech enterprises affected the degree of coupling of the composite system. The former reflects the people’s material and cultural living standards as well as the scale of economic market development during the research period. The Chinese economy was dominated by the market supplemented by government intervention. To stimulate domestic demand, the Chinese government strengthened structural reforms of supply and measurement, where the latter reflects the level of scientific and technological development.

#### 5.3.2 Analysis of factors controlling degree of coupling of composite systems in different regions

The five most influential factors for the degree of coupling of composite systems were efficiency of technical innovation, high-tech product export value, gross domestic product, foreign direct investment, and total retail sales of social consumer goods in the eastern region. Their impact coefficients were all higher than 0.6, and all passed the 0.01 significance level test. The first were most important for the degree of coupling of the composite system. There was confluence of technical talent, perfect transportation construction, and significant scale and cluster effects of high-tech enterprises. Foreign trade developed rapidly in the eastern coastal areas, and the external sales of goods brought advanced foreign technology that promoted innovation. The gross domestic product and foreign direct investment affected the degree of coupling of the composite system. Rapid industrialization accelerated economic development, but has also led to a wide gap between the rich and the poor. In 2015, China utilized a foreign capital of USD 1262.67 trillion, which is the world’s largest foreign investment. It was especially rather large in the eastern region. The total retail sales of social consumer goods also affected the degree of coupling of composite systems. The income of residents has steadily increased, and this supports the increase in the social consumer goods in the eastern region.

The five most influential factors for the degree of coupling of composite system were gross domestic product, efficiency of technological innovation, research and development expenditure, high-tech product export value, and total retail sales of social consumer goods in the central region. Their influence coefficients were higher than 0.6, and all passed the 0.01 significance level test. The gross domestic product had a significant influence on the degree of coupling of composite systems. In recent years, many industries have transferred from the eastern to the central region, and local governments have carried out policy adjustments and institutional innovation to promote economic upgrade and technological innovation. The efficiency of technological innovation, research and development expenditure, and high-tech product export value affect the degree of coupling of composite systems. The resource endowment and industrial foundation region were good in the central areas, government support had improved, and the high-tech industry was consequently developing rapidly. The total retail sales of social consumer goods also affected the degree of coupling of composite systems. The total retail sales of consumer goods is an important part of the GDP, and influences the degree of coordination of the composite system by affecting economic growth.

The five most influential factors for the degree of coupling of composite systems were the number of high-tech companies, number of college students per 10,000, research and development expenditure, number of research and development personnel, and efficiency of technological innovation in the western region. Their influence coefficients were higher than 0.6, and all passed the 0.01 significance level test. The first three strongly affected the degree of coupling of composite systems. The western region had undertaken backward production capacity in the eastern region, but traffic construction was not optimal, educational resources were scarce, and financial support from the government was limited. The number of research and development personnel affected the degree of coupling of composite systems. The delay in educational development led to a lack of technical talent, and few skilled people. The efficiency of technological innovation impacts the degree of coupling of composite systems. Resources are limited, the output of and investment in technological innovation are low, and the efficiency of innovation needs to be further improved in the western areas.

The five most influential factors for the degree of coupling of composite systems were research and development personnel, research and development expenditure, efficiency of technological innovation, gross domestic product, and total retail sales of social consumer goods in the northeast areas. Their impact coefficients are higher than 0.6, and all passed the 0.01 significance level test. The number of research and development personnel significantly affected the degree of coupling of composite systems. The northeastern region had the highest population outflow in China. A large number of technical talents migrated to the Beijing–Tianjin region, resulting in a sharp decline in the number of research and development personnel in the region. Research and development expenditure, efficiency of technological innovation, and gross domestic product also impacted the degree of coupling of composite systems. The national northeast revitalization strategy has led to heavy investment and policy support, but the efficiency of resource allocation remains low, resulting in low efficiency of technological innovation. Affected by the old industrial economic model, the northeastern economy has experienced a significant decline.

#### 5.3.3 Factors analysis of degree of coupling of provincial composite systems

The interaction of two elements had a greater influence than a single factor on the degree of coupling between technological innovation and the regional economic composite system, indicating that a single factor had a positive influence on the degree of coupling of the composite system, and synergy was strong. Research and development expenditure, and the efficiency of technological innovation had the highest combined impact on the degree of coupling of composite systems, which indicates that the two had the strongest interaction with it. Research and development expenditure significantly enhanced the degree of coupling of composite systems by increasing the scale effect of technological innovation. The efficiency of innovation also significantly enhanced this by promoting the high-quality development of the regional economy. The high-tech product export value, effective invention patents, number of college students per 10,000, and amount of foreign direct investment had nonlinear interactions with the degree of coupling of composite systems, and their interactions were significant. The remaining elements were two-factor enhanced interactions, and the effects were not prominent.

## 6. Conclusions and discussion

This paper used the Malmquist index to calculate the total factor productivity and it’s decomposition index of high-tech industries in 30 provinces (cities, districts) in China from 2006 to 2016, and analyzed their temporal and spatial evolution. The authors used the dynamic coupling model to calculate the coupling degree between the efficiency of technological innovation in the high-tech industry and the regional economic level, and used the geo-detector method to analyze the main factors controlling the coordination effect of the composite system. The following conclusions and suggestions can be arrived at:

First, the decline in the total factor productivity occurred mainly due to that in the efficiency of technological progress. The eastern region is rich in resources, but its efficiency of resource allocation is low. We should increase the intensity of research and development, give full play to the scale effect of resource elements, and focus on the development of high-tech industries. This can help form a two-way complementary mechanism of “independent innovation”, “original innovation”, “integrated innovation”, and help use multi-agent integration and collaborative innovation. The decline in technical efficiency occurred mainly due to that in scale efficiency. National and regional governments should expand the scale of high-tech industries in six provinces—Shaanxi, Hainan, Heilongjiang, Yunnan, Jiangxi, and Hebei—especially in the latter three. These regions should give full play to the scale effect, and should promote technological and knowledge innovation at the same time. The decline in the technical efficiency of Heilongjiang occurred due to that in pure technical efficiency. The development model of traditional industries is backward in the northeast. Although China has invested a considerable amount of resources in the industrial upgrade and optimization of the northeast, industrial technology has improved only slowly, and advanced technologies, equipment, and patents have not been fully utilized. This is mainly owing to a lack of matching industrial scale and high-quality talent, which caused the regional efficiency of technological innovation to be low. For this reason, the northeast should formulate the relevant policies, establish a talent pool, attract, retain and use innovative personnel.

Second, the coordination degree of China’s provincial high-tech industry and regional economic complex system are mainly the state of interactive development, and a few are the regeneration stage. In recent years, with the rapid development of the economy and the increase in government support, China’s high-tech industry has developed rapidly, and has a strong synergistic effect with the regional economy. Except in seven provinces—Liaoning, Jilin, Heilongjiang, Shanghai, Jiangsu, Guizhou, and Ningxia—the degree of coupling of composite systems showed an increasing trend overall. In 2016, the composite systems of all provinces were in stages of interactive development. The government should pay attention to the region balanced development while improving the level of high-tech industries and regional economies. It is a long-term and complex task to promote the harmonious and mutual development of composite systems. All provinces should target the key factors affecting the coordinated development of composite systems in the regions, scientifically analyze the core issues influencing regional development and improve and upgrade constraints to enhance the level of coordinated evolution of regional composite systems to regional green growth and sustainable development.

Third, the coupling degree of China’s high-tech industry and regional economic composite system presents the spatial pattern of “Center > East > West > Northeast”. Central and local governments should establish a collaborative innovation system for the high-tech industry nationwide, and introduce policies to accelerate the transfer of high-tech industries and talent in the central, western and northeastern regions. They should seek to eliminate restrictions of geographical proximity on high-tech knowledge spillovers to create a path for high-tech knowledge overflow. The eastern region should improve its marketization and interest compensation mechanisms for high-tech industries, avoid the problem of “external congestion” in the duplication of these to avoid wasting resources, and improve the efficiency of resource allocation for high-tech research and development. The high-tech industries in all regions should give full play to their own advantages, and should optimize the environment for technological innovation and the transformation of results in high-tech industries. They should strive to build a high-tech innovation system with clear division of labor within and between regions for promoting knowledge spillovers, resource sharing, and collaborative innovation in research and development to achieve simultaneous development goals with high collaborative relevance and strong market liquidity.

Fourth, the five most influential factors for the coupling degree in China’s provincial complex system were the regional GDP, efficiency of technological innovation, research and development expenditure, total retail sales of social consumer goods, and the number of high-tech enterprises. Since the reform and opening up, China’s regional economy has recorded unprecedented achievements, but the economy quality remains poor and regional differences are significant. To promote the high-quality development of the regional economy and optimize the economic structure, the Chinese government has implemented supply-side structural reforms, and has promoted the Belt and Road Initiative. Introducing domestic and going abroad develop together. To achieve a balanced development of the region, the Chinese government has introduced a strategy for rural revitalization, increased investment and special poverty alleviation funds, accelerated the development of old-age services. In 2012, the Chinese government proposed an innovation-driven strategy to lead the industry in transferring and upgrading through innovation. To enhance the synergy and aggregation effects between technological innovation and regional economy, the Chinese government is promoting “mass entrepreneurship and innovation” to accelerate the efficiency and rate of technological transformation.

## Supporting information

S1 File (DOC)
