# Supplementary material for: Synergy effects and it’s influencing factors of China’s high technological innovation and regional economy
Source: PLoS One. 2020 May 20;15(5):e0231335. doi: 10.1371/journal.pone.0231335 (PMC7239604; doi:10.1371/journal.pone.0231335)
Supplement: S1 File — (DOC) [file pone.0231335.s001.doc]

**Highlights**

1. Total factor productivity showed a "W"-type trend, others exhibited a volatile trend.
2. The Total factor productivity formed a core-edge spatial pattern.
3. Coupling degree exhibited spatial pattern of Central > Eastern > Western > Northeastern.
4. Economy level, technology innovation, research expense affected coupling degree mostly.
